# Curcumin inhibits the neuroimmune response mediated by mast cells after pulpitis

**DOI:** 10.1590/1678-7757-2023-0456

**Published:** 2024-08-30

**Authors:** Ming DONG, Jing TANG, Lu-Jia LI, Ting DAI, Yi-Yan ZUO, Hai-Wei JIN

**Affiliations:** 1 Dalian Medical University School of Stomatology Dalian China Dalian Medical University, School of Stomatology, Dalian, China.; 2 Suqian Stomatological Hospital Suqian China Suqian Stomatological Hospital, Suqian, China.; 3 Dalian Medical University The Affiliated Stomatological Hospital Dalian China Dalian Medical University, The Affiliated Stomatological Hospital, Dalian, China.

**Keywords:** Pulpitis, Mast Cells, Curcumin, Neuroimmune

## Abstract

**Objective:**

To analyze the effect of mast cells (MCs) in neurogenic inflammation and the neuroimmune response of trigeminal ganglia (TG) due to pulpitis and detect the regulatory effect of curcumin (Cur) on neuroimmune responses induced by pulpitis.

**Methodology:**

Immunohistochemistry, toluidine blue staining (TB), and other methods were used to detect the dynamic changes of MCs, as well as tryptase expression changes and protease activated receptor 2 (PAR2) and calcitonin gene-related peptide (CGRP) levels in the neuroimmune response induced by pulpitis. After administering Cur by intraperitoneal injection, the expression levels of Toll-like receptor 4 (TLR4), CGRP, glial fibrillary acidic protein (GFAP), fractalkine (CX3CL1), Tumor necrosis factor (TNF-α), and other factors were examined in the TG of pulpitis-induced rats.

**Results:**

After pulpitis induction, the expression of CGRP-positive neurons and GFAP-positive soluble guanylate cyclase (SGC) in the TG significantly increased. A large number of MCs underwent degranulation. MCs were scattered between the CGRP-positive nerve fibers. MCs showing a typical degranulated state within the TG significantly increased and tryptase-positive MCs surrounded the TG nerve fibers and neurons. After treatment with Cur, the inflammatory response in the periodontal bone induced by pulpitis decreased and promoted early tissue repair. The expression of TNF-α significantly decreased as did degranulation of MCs. In contrast, the expression of CGRP, TLR4-positive neurons, activated SGCs, and PAR2-positive TG neurons significantly decreased. MCs could participate in the neuroimmune response induced by pulpitis by the tryptase signaling pathway.

**Conclusion:**

Importantly, Cur inhibited the degranulation of MCs, downregulated the expression of tryptase and PAR2 in the TG, and attenuated the activation response of osteoclasts in the apical periodontium.

## Introduction

Pulpitis is characterized by an immune response that involves various immunoglobulins, mast cells (MCs), and complement components, suggesting that antibody-mediated immune reactions occur in the dental pulp and periapical tissues.^1,2^ MCs are a type of versatile immune cell that contains various bioactive substances in their cytoplasmic granules. When activated, they can degranulate and release multiple mediators that participate in various physiological and pathological processes.^3,4^ As a key effector cell in the inflammatory process, MCs can interact with neurons and serve as an important link between the nervous and immune systems.^5^ The form of degranulation release mediators of MCs is variable. For example, activation of the FcεR1 receptor can cause large particles of MCs to slowly and continuously release inflammatory mediators, and through the activation of Mas-related G-protein coupled receptor member X2, it can result in a rapid and short-lived release mediators of small particles.

In recent years, the role of neuroimmune mechanisms in pulpitis has become a focus of the attention of research.^6,7^ During degranulation, MCs release prestored mediators and a large number of neuropeptides, cytokines, and other substances that include tryptase, tumor necrosis factor-alpha (TNF-α), and nerve growth factor, which affects neuronal function.^8,9^ Tryptase is a serine protease that is an extremely abundant medium in MCs granules and is the main secreted protein during MC degranulation. Its high expression is a typical sign of MC activation.

Peripheral neurons react to this stimulation and release many neuropeptides to regulate the activation of MCs, including the calcitonin gene-related peptide (CGRP), which plays a role as a pain factor in the nervous system, transmitting nociceptive sensations.^10,11^ In turn, CGRP can directly activate MCs by the CGRP receptor. Furthermore, tryptase from MCs can activate protease-activated receptor-2 (PAR2) in the trigeminal ganglia (TG), leading to the release of nitric oxide, substance P, and CGRP.^12-15^

Curcumin (Cur) is a polyphenolic compound in turmeric, which belongs to the ginger family.^16,17^ Cur possesses antioxidant and anti-inflammatory properties and has widely served to treat various diseases.^18^ Studies suggest that Cur can exert anti-inflammatory, neuroprotective, and immune-regulatory effects by different mechanisms. Based on those findings, we speculated that Cur may configure a promising drug to treat neurogenic inflammation.^19,20^

Pulpitis could induce the neuroimmune response of TG. We hypothesized that the TG had MCs that would show an obvious degranulation state after the occurrence of pulpitis. We also wanted to find the neuroimmune mechanism of MCs involved in the induction of pulp inflammation. Here, we used Freud’s Complete Adjuvant (CFA) to establish an animal model of experimental pulpitis in rats to investigate the role of MCs and the tryptase pathway in neurogenic inflammation induced by pulpitis and the associated therapeutic effects of Cur.

## Methodology

### Establishment of an experimental pulpitis animal model

A total of 84 adult male Sprague-Dawley rats weighing 200–230 g were provided by the Special Pathogen Free Animal Experiment Center of Dalian Medical University (Animal Ethical Approval Number: AEE19020). The rats were divided into two groups: a pulpitis group and Cur group, with 42 rats in each group. Each group was further divided into seven time points: 0, 24, and 72 h, and 1, 2, 4, and 6 weeks; with six rats used at each time point. Among them, half the rats from each group were used for immunohistochemistry, whereas the other half were used for western blotting experiments. The pulpitis model was induced in the mandibular first molar after exposure of the pulp cavity and insertion of a small cotton ball containing 5 µL of CFA emulsion (CFA:saline = 1:1) into the pulp cavity, after which the cavity was temporarily sealed with a glass-ionomer cement. Following establishment of the model in the Cur group, Cur (dissolved in 5% DMSO, diluted with olive oil and prepared as an injection solution at a concentration of 25 mg/mL) was injected into the abdominal cavity at a dose of 100 mg/kg, once every 24 h for a maximum of four consecutive injections depending on the sampling time.

### Western blot analysis

Proteins from endometrial tissues or cells were extracted with lysis buffer (KeyGen Biotech Co., Ltd., Nanjing, China) following the manufacturer’s instructions. The concentration of the protein extracts was then determined using a protein quantitative kit (TransGen Biotech, Beijing, China), after which 40 µg of total protein was denatured in 6× protein loading buffer for 7 min at 100°C. The proteins were then separated by electrophoresis using 8% sodium dodecyl sulfate-polyacrylamide gel electrophoresis and transferred onto a nitrocellulose membrane (Millipore, Bedford, MA, USA) by electroblotting for 90 min at 4°C. The membranes were then blocked with 5% nonfat dried milk in Tris-buffered saline (10 mmol/L Tris, pH 7.5 and 0.14 mol/L NaCl) with 0.1% Tween 20 (TBST) for 2 h at room temperature followed by incubation with the appropriate primary antibody overnight at 4°C. The following antibodies were used: anti-CX3XL1 (diluted 1:1,000; Abcam, Cambridge, MA, USA), anti-PAR2 (diluted 1:100; Abcam), and anti-GAPDH (diluted 1:1,000; Abcam). The following day, the membranes were incubated with the appropriate secondary antibody (diluted 1:500; ABclonal, Woburn, MA, USA) for 1 h. ECL luminescent solution was employed to visualize the immunoreactive bands on a Bio-Rad gel imaging system (Bio-Rad, Hercules, CA, USA) and the results analyzed using Image Lab.

### Immunohistochemistry

The tissue was sectioned at 4-μm intervals and baked for ٦٠ min at ٥٦℃. After deparaffinization and rehydration of the tissue sections, antigen retrieval was performed for 20 min at 100℃ in citrate buffer, followed by 3% peroxide treatment for 15 min at room temperature in the dark to quench endogenous peroxidase. The sections were then washed in phosphate-buffered saline and blocked in 5% goat serum for 15 min at 37°C to prevent non-specific binding before being incubated with the following primary antibodies: anti-CGRP (diluted 1:5,000; Abcam), anti-TLR4 (diluted 1:500; Abcam), anti-TNF-α (diluted 1:200; Santa Cruz Biotechnology, Santa Cruz, CA, USA), anti-GFAP (diluted 1:300; Santa), and anti-tryptase (diluted 1:200; Abcam). The sections were incubated with biotinylated secondary antibody (ZSGB-Bio Co., Ltd, Beijing, China) for 30 min at 37°C. After washing again, the sections were incubated with streptavidin-horseradish peroxidase (ZSGB-Bio) for 30 min at 37°C. Positive immunoreactions were visualized with diaminobenzidine-peroxidase substrate (ZSGB-Bio), after which the sections were counterstained with hematoxylin and eosin for 30 s, dehydrated, and mounted in distrene dibutylypthalate xylene.

### Immunofluorescence staining

Frozen tissue sections were air-dried for 30 min at 37℃, rinsed three times with phosphate-buffered saline, incubated in an 80% methanol solution containing 0.3% H_2_O_2_ at room temperature for 60 min, followed by incubation with normal donkey serum for 60 min at room temperature. CGRP (1:5000) or GFAP (1:300) or TNF-α (1:200) antibodies were added. DAPI (1ug/mL) working solution was added in the dark for nuclear background staining.

### Toluidine blue staining

Slices were stained in toluidine blue staining solution for 20 min, then placed in 95% ethanol, dehydrated in 100% ethanol, cleared with xylene, and sealed with Hamsan gum.

### Hematoxylin-eosin (HE) staining

The slices were dried at a constant temperature of 37℃ for 30 min. Then, 300 μL of hematoxylin staining was added, and the staining was performed for 20 min. The slides were stained with eosin for 2 s, cleared with xylene, and sealed with Hamsan gum.

### TRAP staining

To perform TRAP staining, the cells were first fixed by adding 1 mL of 4% paraformaldehyde to the cells and incubating them for 20 minutes at room temperature. The TRAP staining solution was prepared and incubated with the cells at 37℃ for 1.5 hours. Finally, hematoxylin was added for a 2-minute re-staining step. The stained cells were observed and images were captured under an inverted microscope.

### Statistical analysis

All data were analyzed and are shown as means ± standard deviations (SD). One-way ANOVA was performed and plotted using SPSS 13.0 for Windows (SPSS, Chicago, IL, USA), and differences were considered statistically significant at P values < 0.05.

## Results

### The histopathological manifestations of pulpitis induced by CFA

**Figure 1:**
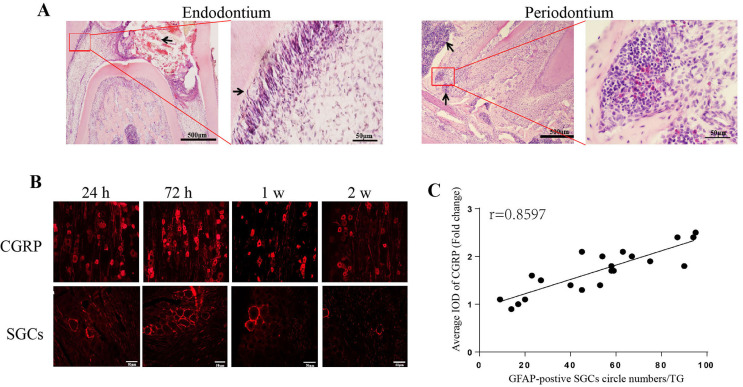
The histopathological manifestations of pulpitis induced by CFA. A: HE staining results of CFA-induced pulpitis. Inflammatory cells occurred in the endodontium and periodontium (indicated by arrows), (Scale bar = 500 μm; Scale bar = 50 μm). B: CGRP-positive neurons and GFAP-positive SGCs in the TG. (Scale bar = 50 μm). C: Results of CGRP and GFAP correlation analysis.

The results of HE staining showed a necrotic area in the coronal pulp with the opening of the pulp as the center 24 h after modeling (Figure 1-A). The arrangement of dental pulp cells was disordered and odontoblasts underwent vacuolar degeneration. Many plasma cells and neutrophils occurred below the apical foramen, spreading to the surrounding alveolar bone. Overall, results indicated the successful construction of a rat model of pulpitis induced by CFA.

Immunofluorescence staining indicated CGRP-positive TG neurons and GFAP-positive SGCs. CGRP was expressed in the cytoplasm of large, medium, and small neurons, whereas SGCs formed a ring-like structure surrounding the neurons. At 24 h after pulpitis induction, the expression of CGRP-positive neurons and GFAP-positive SGCs in the TG significantly increased, reaching a peak at 72 h and lasting for six weeks (Figure 1-B). A significant positive correlation occurred (Pearson’s correlation coefficient r = 0.8597, p < 0.0001) between CGRP-positivity and SGCs (Figure 1-C).

### The expression of MCs and CGRP-positive neurons induced by pulpitis

**Figure 2:**
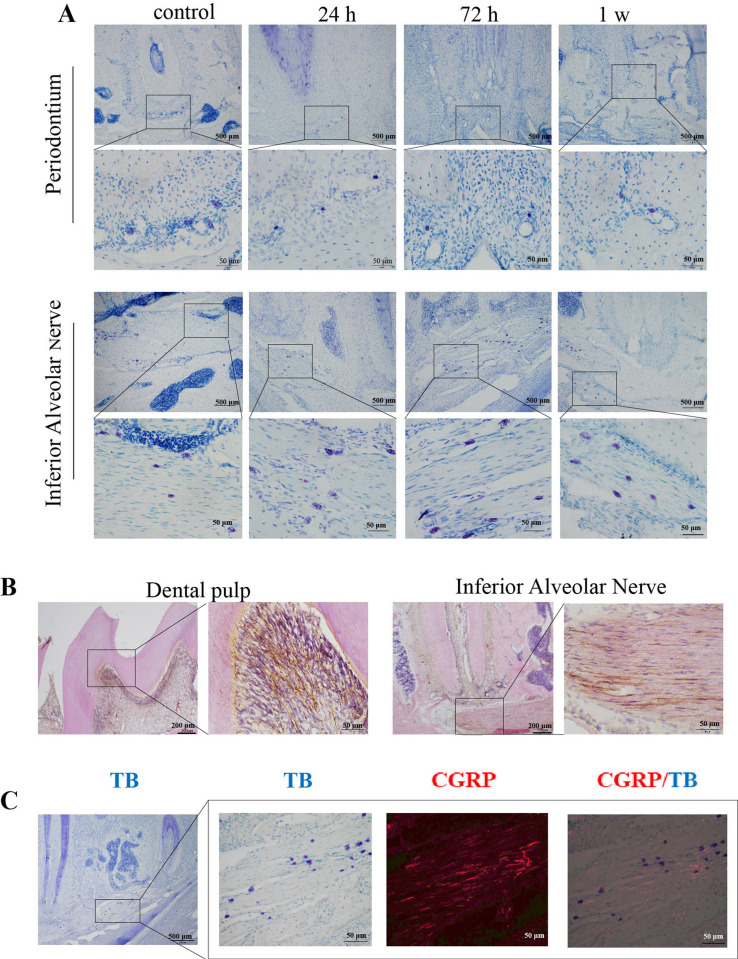
MCs and CGRP expression in the periodontium and inferior alveolar nerve after pulpitis. A: T toluidine blue staining of MCs in the periodontium and inferior alveolar nerve (scale bar = 50 and = 200 μm). B: CGRP and HE double staining (scale bar = 200 and 50 μm). C: CGRP staining and toluidine blue double labeled MCs (scale bar =50 and 500 μm).

After inducing pulpitis in the right mandibular first molar, this study found no obvious sign of MC degranulation in the periodontium. However, many MCs occurred in the inferior alveolar nerve. In the control group, MCs in the inferior alveolar nerve showed intact cell membranes without obvious degranulation. After 24 h after pulpitis induction, a large number of MCs underwent degranulation, which persisted for further 48 h. At one week after pulpitis induction, the number of MCs that underwent degranulation decreased (Figure 2-A).

CGRP immunohistochemistry and HE double staining (Figure 2-B) showed many CGRP-positive nerve fibers in the dental pulp and inferior alveolar nerve of the teeth. Immunofluorescence staining and TB showed a close anatomical association between CGRP-positive nerve fibers in the inferior alveolar nerve and MCs. MCs were scattered between the CGRP-positive nerve fibers and occurred near them. This study found few toluidine blue-labeled MCs in the dental pulp chamber (Figure 2-C).

### Dynamic expression of MCs and related factors induced by pulpitis

**Figure 3:**
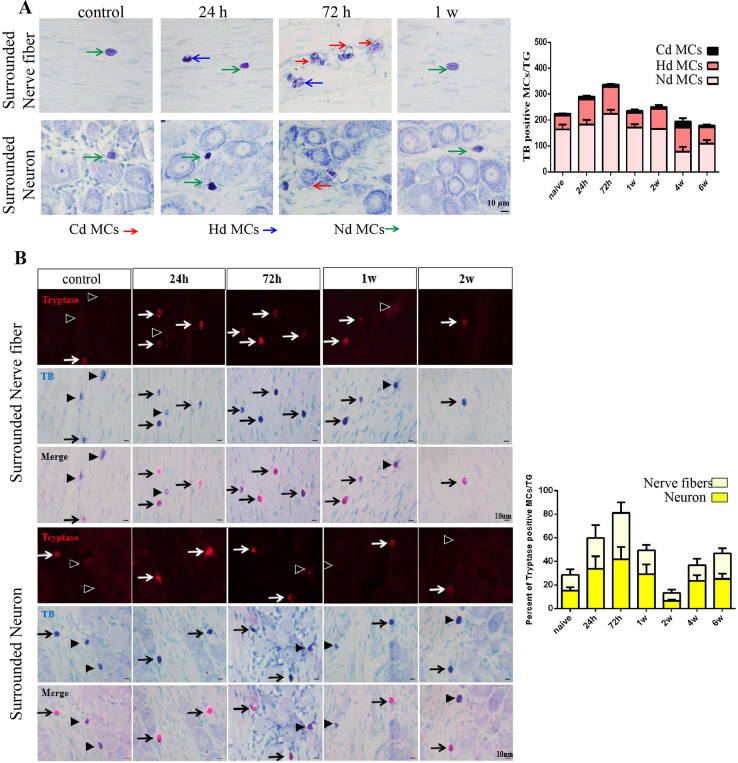
Dynamic expression of MCs and related factors induced by pulpitis. A: Toluidine blue staining showed that MCs surrounded TG neurons and nerve fibers (scale bar = 10 μm). B: Tryptase and toluidine blue staining indicated tryptase-positive MCs in the TG nerve fibers and surrounding neurons (scale bar = 10 μm).

We categorized MCs cells into three states: complete degranulated MCs (Cd MCs), half-degranulated MCs (Hd MCs), and non-degranulated MCs (Nd MCs). TB results showed that MCs surrounded TG neurons and nerve fibers (Figure 3-A). MCs were round or oval, with intact cell membranes and a cytoplasm that was rich in dense purple-stained granules. Cell diameter totaled about 10-25 μm. Pulpitis induction significantly increased MCs, showing a typical degranulated state within the TG. The cell membrane of MCs was completely or partially absent, and the granules inside the cell were diffusely scattered. The statistical analysis showed that at 24 h after pulpitis induction, the total number of MCs (Cd, Hd, and Nd MCs) per unit area in the TG significantly increased, reaching a peak at 72 h after pulpitis induction before returning to normal after one week. At the same time, the number of Cd MCs in the TG significantly increased, reaching a peak at 72 h after pulpitis induction before returning to normal levels.

Tryptase immunofluorescence staining and TB showed tryptase-positive MCs around TG nerve fibers and neurons (Figure 3-B). Immunofluorescence and TB results showed the coexistence of tryptase-positive and MCs within the TG. The statistical analysis of the tryptase-positive cell ratio (tryptase-positive MCs/TB-positive MCs) showed a significant increase in the tryptase-positive cell ratio at 24 h after pulpitis induction, reaching a peak at 72 h before returning to normal levels after two weeks.

### The pathological manifestations of pulpitis after Cur treatment

**Figure 4:**
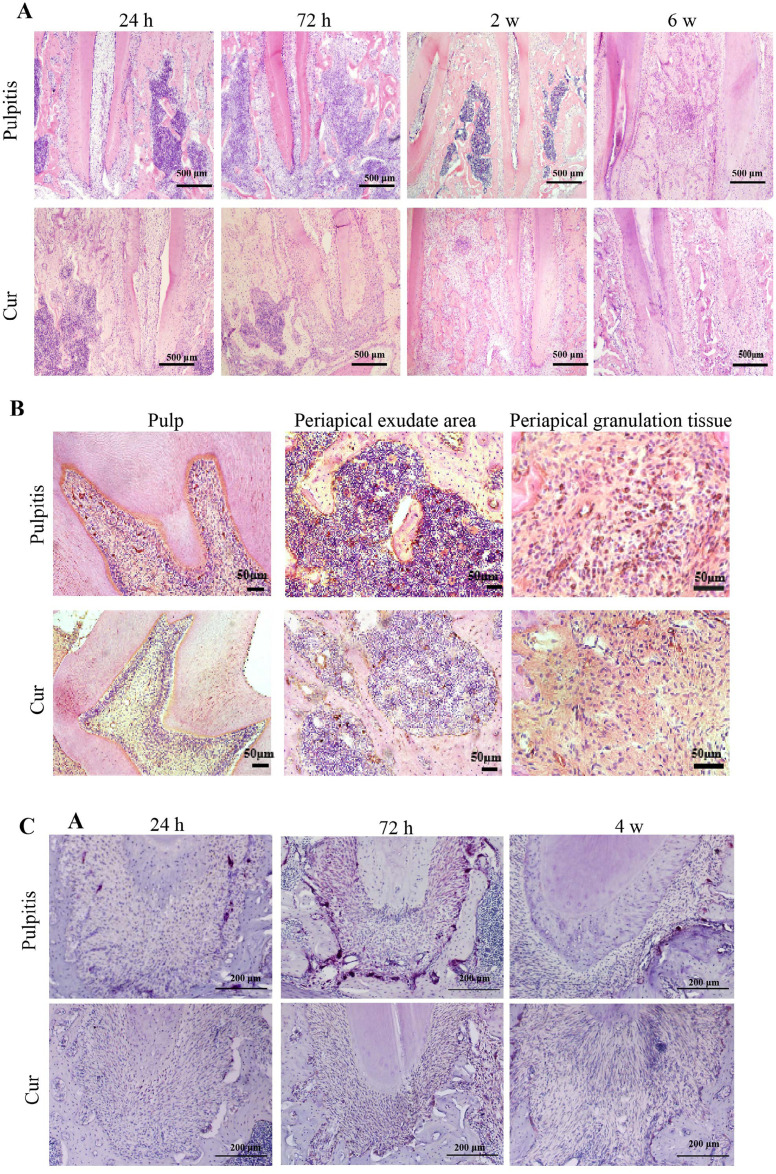
The pathological manifestations of pulpitis after Cur treatment.

HE staining results showed that at 24 h after pulpitis induction, many inflammatory cells infiltrated the periodontal bone around the apical foramen of the mandibular first molar, which continued for four weeks. After six weeks, the exudation area around the apical foramen was gradually covered by fibrous tissue. However, Cur treatment significantly decreased the degree of inflammatory exudation in the periodontal bone around the apical foramen at 72 h, causing fibrous formations after two weeks and the gradual disappearance of the exudation area. Results suggested that Cur reduced the inflammatory response in the periodontal bone around the apical foramen induced by pulpitis and promoted early tissue repair (Figure 4-A).

A: HE staining results after Cur treatment (scale bar = 500 μm). B: Immunohistochemistry and HE staining results after Cur treatment (scale bar = 50 μm). C: TRAP staining results after Cur treatment (scale bar = 200 μm).

Immunohistochemistry staining results showed that at two weeks after pulpitis induction, the pulp had many TNF-α-positive cells. In contrast, the Cur group showed a significant reduction in TNF-α positive cells in the pulp chamber. Specifically at 72 h after pulpitis induction, the periapical exudate area showed a significant presence of TNF-α positive cells, whereas TNF-α expression noticeably decreased in the Cur group. At six weeks after pulpitis induction, many TNF-α positive cells still occurred in the periapical granulation tissue, whereas the Cur group showed a significant reduction in TNF-α expression (Figure 4-B).

TRAP staining results showed that, at 24 h after pulpitis induction, the periapical alveolar bone showed few activated osteoclasts. However, at 72 h after pulpitis induction, the periapical alveolar bone showed many activated osteoclasts, which mainly occurred at the junction of the periodontal ligament and alveolar bone. The activation of osteoclasts lasted up to two weeks after pulpitis induction. However, in the Cur treatment group, the number of activated osteoclasts significantly decreased at the same time points (Figure 4-C).

### Cur inhibited the expression of MCs and associated factors in TG neurons

**Figure 5:**
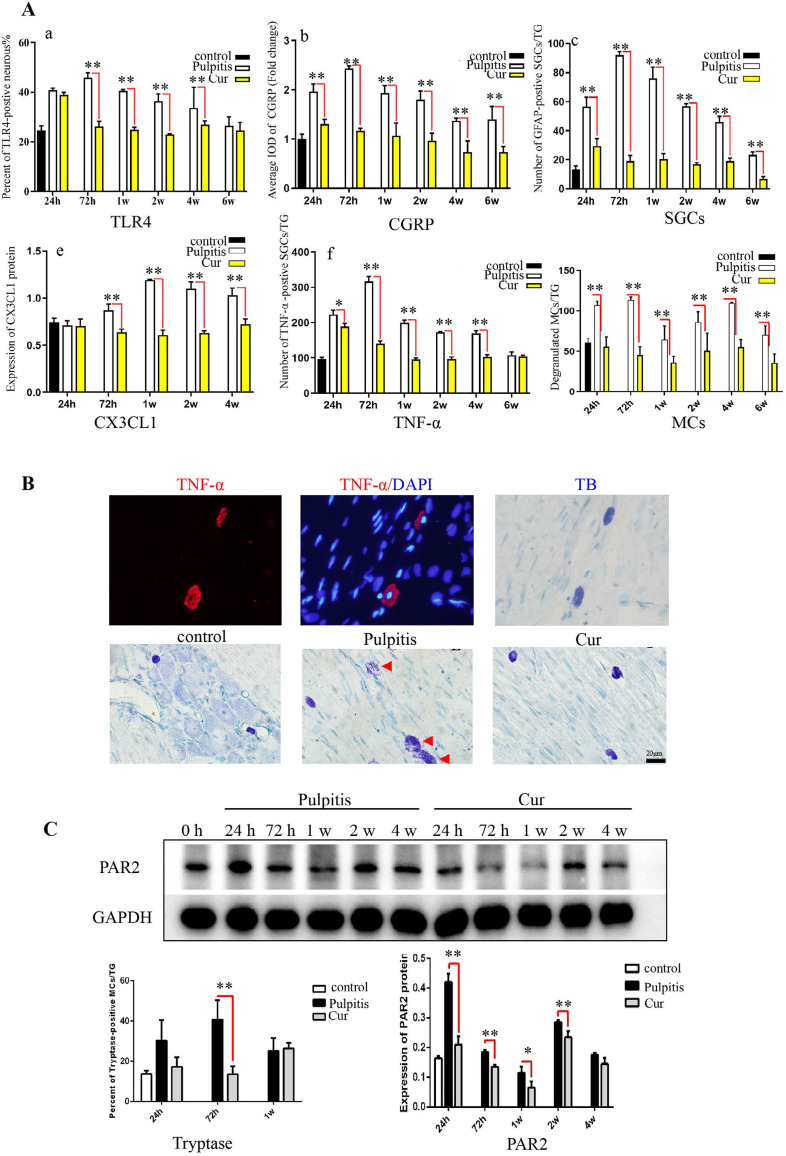
Cur treatment inhibited the expression of MCs and associated factors in TG neurons. A: Statistical analysis results of TLR4-positive neurons, CGRP-positive neuronal fluorescence optical density values, activated SGC counts, CX3CL1 expression statistics, TNF-α-positive cell counts, and MC counts. B: Morphology and distribution of TG mast cells labeled by toluidine blue staining (scale bar = 20 μm). C: Western blot results of PAR2 expression (**p < 0.01, *p < 0.05).

At 24 h pulpitis induction, the number of TLR4-positive neurons significantly increased when compared with controls, reaching a peak at 72 h and lasting for four weeks. However, in the Cur group, from 72 h up to four weeks after pulpitis induction, the number of TLR4 positive neurons in the TG significantly decreased when compared with the control group. At 24 h after pulpitis induction, the number of CGRP-positive neurons in the TG significantly increased when compared with controls, reaching a peak at 72 h and lasting for fix weeks. However, in the Cur group, the number of CGRP-positive neurons in the TG significantly decreased. Similarly, the number of activated SGCs in the TG significantly increased after pulpitis induction, reaching a peak at 72 h and lasting for six weeks. However, in the Cur group, the number of SGCs significantly decreased at the same time points. CX3CL1 in the TG significantly increased at 72 h after pulpitis induction, reaching a peak at one week and lasting for four weeks. However, in the Cur group, CX3CL1 was significantly inhibited at the same time points. The number of TNF-α-positive cells in the pulpitis significantly increased at 24 h after pulpitis induction, reaching a peak at 72 h and lasting for four weeks. However, in the Cur group, the significant increase in TNF-α positive cells failed to occur at the same time points. Moreover, the number of degranulated MCs significantly increased at 72 h after pulpitis induction, which lasted for four weeks. However, the application of Cur treatment significantly inhibited this increase in degranulated MCs at the same time points (Figure 5-A).

TB results for MCs in the TG showed that TNF-α expression primarily occurred in enlarged toluidine blue-positive MCs (Figure 5-B). Moreover, TB results for TG showed that after pulpitis induction, some of the enlarged MCs had ruptured cell membranes, causing blue-purple particles in the cytoplasm to be released from the cells. The cells lost their original morphology and clusters of blue-purple particles showed a bursting pattern. However, after Cur treatment, the number of degranulated MCs significantly decreased. Western blot results (Figure 5-C) indicated a significant increase in PAR2 expression at 24 h until two weeks after pulpitis induction. However, Cur treatment effectively reduced the expression of PAR2 at the same time points.

## Discussion

Recent studies had shown that nerve infections activate resident immune cells and glial cells, recruiting immune cells in the circulation to respond to the injury and causing the nerve endings at the immune stress response at the injury site to become more sensitive. The dental pulp tissue is rich in nerve fibers and blood vessels. When the dental pulp is injured, nerve fiber immune cells from TG primary sensory neurons communicate with each other. Therefore, pulpitis can also be called a “neurogenic inflammation,” and neuroimmune intervention and regulation may be a new way to treat pulp inflammation and pain. MCs are a type of multi-functional immune cells commonly occurring around blood vessels, lymph, and nerves.^21,22^ The cytoplasmic granules of MCs contain various bioactive substances, such as tryptase and TNF. MC activation causes degranulation and releases multiple factors, many of which are involved in various physiological and pathological processes.^2,23^ Studies have shown that high expression of tryptase is a typical indicator of MC activation.^24^ Interestingly, tryptase-positive MCs have been found in the alveolar bone marrow of female estrogen-deficient diabetic rats and in periapical granulomatous in humans, indicating the involvement of activated MCs in related inflammatory responses.^25,26^ Therefore, this experiment used tryptase as a marker for activated MCs to investigate the role and possible mechanisms of MCs and the tryptase pathway in neurogenic inflammation and TG neuroimmune responses induced by pulpitis.

MCs show heterogeneous anatomical location, microenvironment, and species.^27,28^ This study found that a variability in the morphology and size of toluidine blue-labeled MCs in the periodontal membrane as well as in the inferior alveolar plexus. For example, MCs in the periodontium had lighter cytoplasmic staining than that of the control group, with diameters ranging from 17 to 24 μm. Furthermore, their cytoplasmic granules were more deeply stained in the pulpitis, ranging from 8 to 15 μm. In contrast, the distribution of MCs in the inferior alveolar nerve plexus varied considerably. The control group showed many MCs with sizes measuring more than 20 μm in the sheath membrane of the inferior alveolar nerve but few MCs in the nerve fibers. Moreover, after pulpitis, many MCs migrated into the nerve fibers. The diameter of MCs in the TG totaled about 10-25 μm, and the cell volume was significantly larger than that of MCs in periodontal membrane and bone marrow cavity.

We also found that the cytoplasmic staining of MCs with toluidine blue in the dental pulp was light, and it was difficult distinguish these MCs from the surrounding fibroblasts. The light staining may be due to the tissue damage associated with pulpitis and the release of neuropeptides such as CGRP secreted by nerve fibers after pulpitis, which induced degranulation of MCs by CGRPR. Therefore, the cytoplasmic staining was unclear, which affected the further analysis and study of MCs in the pulp cavity. Nigrovic discovered that the synovium of patients with rheumatoid arthritis significantly increased the number of MCs. The increased number of MCs in turn initiated an inflammatory response that increased^29^ vascular permeability, recruitment of white blood cells, and activation of resident synovial fibroblasts and macrophages. This study found that the number of MCs in the TG significantly increased after pulpitis. Immunohistochemical staining for tryptase showed that MCs were more densely distributed around the nerve fibers of the maxillary branch of the trigeminal nerve at 24 h, 4 weeks, and 6 weeks after pulpitis. At 72 h, 1 week, and 2 weeks after pulpitis induction, the number of MCs around the nerve fibers near the ocular branch significantly increased. Results suggested that pulpitis triggered a neuroimmune response in the trigeminal ganglion, leading to the migration or recruitment of MCs, possibly from other tissues such as the blood, which included circulating MC progenitor cells. After a significant increase in the degranulation of MCs, levels returned to normal because of the recruitment of the MC progenitor cells in the blood, division of resident MCs, maturation of local MC precursors, and a decrease in apoptosis of pre-existing MCs. Overall, these processes helped maintain the homeostasis of the microenvironment. Additionally, within 72 h of pulpitis induction, a significant increase in the number of Cd MCs occurred in the TG. Meanwhile, in the dental pulp nerve plexus below the root apex, a large number of Cd MCs appeared within 24 h after pulpitis induction. Results suggested that MCs in the dental pulp nerve plexus, which were closer to the injurious stimuli, rapidly activated and participated in neurogenic inflammatory responses. On the other hand, TG neurons that innervated the dental pulp and other immune cells involved in neuroimmune responses reacted at a later stage, contributing to the regulation of pulpitis inflammation and pathological processes. Tryptase is the most abundant neutral protease in MCs granules, and PAR2 is the specific receptor for Tryptase and trypsin. Steinhoff’s research found that Tryptase of MCs can regulate neuronal activity by activating PAR2.

At present, many methods can prepare rat experimental pulpitis model, such as endotoxin lipopolysaccharide induction, soft caries implantation, high heat stimulation, pulp opening, etc. CFA is an ideal inflammatory substance and inflammatory pain inducer that is widely used in pain research. The experimental pulpitis and pulp inflammatory pain sensitivity model in rats was prepared by opening the pulp and encapsulating CFA, which can more effectively simulate the clinical pulpitis and pain sensitivity process. It also has the characteristics of short modeling time stable animal model and good reproducibility.

Cur is known for its strong antioxidant and anti-inflammatory properties, which makes it highly promising as a disease treatment. However, due to its low water solubility, poor absorption, quick elimination, and rapid metabolism, it is yet to be widely used in clinical treatment. This study used the commonly used intraperitoneal injection method for Cur administration in our pulpitis rat model. The rats were administered Cur via intraperitoneal injection on the same day as pulpitis induction by CFA, after which the treatment was continued for four days. This method helped maintain a stable concentration of Cur in the bloodstream of the rats, enabling a rapid and effective pharmacological effect of Cur. Results showed that after intraperitoneal injection of Cur, the inflammatory exudation in the periapical alveolar bone of the Cur group rats significantly decreased and the formation of ossified tissue advanced significantly in relation to the control group. Results suggested that Cur could effectively decrease alveolar bone resorption.

Studies have shown that the source of TNF-α in inflammatory dental pulp may be released from the degranulation of MCs in oral tissues. The experimental results of this study found that TNF-α was highly expressed on MCs induced by pulpitis, and the application of Cur could significantly reduce the expression of TNF-α. At the same time, studies had shown that reactive oxygen species (ROS) played an important role in MCs degranulation by regulating extracellular calcium influx and mediating histamine release. Therefore, the application of Cur could inhibit the degranulation of MCs and reduce the inflammatory response by reducing the release of ROS. The results of this study confirmed that after CFA induced pulpitis, MCs in the TG showed degranulation; and after the application of Cur, the number of MCs cells in the degranulated state significantly decreased. Cur may regulate the neuroimmune response of TG by inhibiting the degranulation reaction of MCs in TG and participate in regulating the neurogenic inflammatory response of dental pulp.

Multiple studies have shown that TLR4 could directly affect their function and contribute to the formation of neuroimmune network communication signals between neuronal cells and immune cells, thereby maintaining pain hypersensitivity reactions in the central nervous system and peripheral nervous systems.^30^ Research has confirmed that the number of TLR4-positive neurons, mainly CGRP-positive neurons, significantly increased during the plasticity response of neuronal damage receptors induced by pulpitis.^31^ Evidence also suggests that Cur intra-articular administration can significantly downregulate the overexpression of TLR4 and its downstream activation of NF-κB inhibit synovial inflammation and improve articular cartilage damage an arthritic knee induced lipopolysaccharide rat model. This study confirmed that the expression of TLR4 on the cell bodies of TG neurons significantly increased after pulpitis, which was accompanied by increased TNF-α expression and CGRP immunofluorescence intensity in the TG.

However, the Cur treatment significantly reduced the expression of TLR4, CGRP, and TNF-α in the TG, and the expression of TLR4 and CGRP showed a strong correlation. These results suggested that after pulpitis, TG neurons that dominated the pulpitis triggered the immune response via TLR4, further activating other TG neurons.^32^ High expression of CGRP was evident along the nerve fibers to the dental pulp tissue, which likely participated in the inflammation and pain responses.^33^ Cur treatment regulated neuroimmune responses by reducing the expression of TLR4 in the TG, exerting anti-inflammatory, analgesic, and neuroprotective effects. We also showed that the intraperitoneal injection of Cur significantly inhibited the activation response of SGCs induced by pulpitis, reduced the overexpression of neuronal CGRP, and suppressed the release of inflammatory factors in the TG. These findings suggested that Cur modulated neuroinflammation and immune responses by regulating the activation of neurons and SGCs, inhibiting neuroinflammation, reducing pain responses, and regulating peripheral neurogenic pulpitis and pain.^34^ Soluble CX3CL1 within the peripheral tissue may attract natural killer cells, T cells, and macrophages. Local pro-inflammatory cytokines such as TNF-α, IL-1β, and IFN-γ are involved in regulating the synthesis and expression of CX3CL1.^35,36^ Our experimental research showed that Cur treatment significantly reduced the expression of CX3CL1 after pulpitis, preceding a significant decrease in the inflammatory factor TNF-α. Results suggested that Cur participated in the neuroimmune response associated with pulpitis by inhibiting the expression of CX3CL1 within the pulpitis, thereby suppressing neuroinflammation and pain hypersensitivity.

We also found decreased expression of TNF-α within enlarged MCs, which suggested that Cur reduced allergic inflammatory reactions by inhibiting the degranulation of MCs in the TG, reducing neuronal excitability and weakening the neuroimmune response and pain sensitivity induced by pulpitis. The Cur treatment significantly decreased the percentage of tryptase-positive MCs and significantly inhibited the expression level of PAR2. Results further suggested that Cur alleviated the neuroimmune response by reducing the expression of tryptase and PAR2, reducing the excitability of neurons and relieving pain hypersensitivity.

## Conclusions

This study established a rat model of pulpitis to investigate the role and possible mechanisms of Cur in the neuroimmune response induced by pulpitis. Results provided evidence that pulpitis degranulated the MCs in the inferior alveolar plexus and TG. MCs can participate in the neuroimmune response induced by pulpitis by the tryptase signaling pathway. Cur treatment had anti-inflammatory, analgesic, and neuroprotective effects after pulpitis applications by inhibiting the degranulation reaction of MCs, downregulating the expression of Tryptase and PAR2 in TG, which may also be used in immunoregulation and anti-inflammatory therapies.
